# Recent Developments in Food Packaging Based on Nanomaterials

**DOI:** 10.3390/nano8100830

**Published:** 2018-10-13

**Authors:** Yukun Huang, Lei Mei, Xianggui Chen, Qin Wang

**Affiliations:** 1 School of Food and Bioengineering, Xihua University, Chengdu, Sichuan 610039, China; huangyukun@mail.xhu.edu.cn (Y.H.); chen_xianggui@mail.xhu.edu.cn (X.C.); 2 Department of Nutrition and Food Science, College of Agriculture and Natural Resources, University of Maryland, College Park, MD 20740, USA; leimei@umd.edu

**Keywords:** nanomaterials, food packaging, inorganic nanoparticles, organic biopolymer composites, synthesis, activity, application, safety assessment, mechanisms

## Abstract

The increasing demand for high food quality and safety, and concerns of environment sustainable development have been encouraging researchers in the food industry to exploit the robust and green biodegradable nanocomposites, which provide new opportunities and challenges for the development of nanomaterials in the food industry. This review paper aims at summarizing the recent three years of research findings on the new development of nanomaterials for food packaging. Two categories of nanomaterials (i.e., inorganic and organic) are included. The synthetic methods, physical and chemical properties, biological activity, and applications in food systems and safety assessments of each nanomaterial are presented. This review also highlights the possible mechanisms of antimicrobial activity against bacteria of certain active nanomaterials and their health concerns. It concludes with an outlook of the nanomaterials functionalized in food packaging.

## 1. Introduction

Nanoscience and nanotechnology have become exciting fields of research and development since its introduction by Richard Feynman in 1959 [1]. At the heart of research in these fields are the synthesis, characterization, modeling and applications of new materials with nanometer-scale dimensions, at least one of the three external dimensions ranging from approximately 1 nm to 100 nm, which are called “nanomaterials”. There are numerous nanomaterials that have been reported in many prior studies, generally divided into the so-called zero-dimensional (e.g., nanoparticles (NPs): quantum dots, nanoclusters and fullerenes), one-dimensional (e.g., nanotubes and nanorods), two-dimensional (e.g., thin films), and three-dimensional (e.g., nanocomposites and nanofibers) nanomaterials [2]. These materials have exhibited unusual mesoscopic properties, including high surface area, fine particle size, high reactivity, high strength and ductility, which are the reasons that nanomaterials are frequently applied in a diversified range of industrial fields [3,4]. As the researches of multi-disciplinary areas move along, nanomaterials are advancing with wide applications to electronic, optical and magnetic devices, biology, medicine, energy, defense and so on. In addition, their developments in food and agriculture industries are nearly similar to their modernization in medicine delivery and pharmaceutical areas [5,6].

In recent years, owing to the unique properties of nanomaterials other than their bulk counterparts mainly covering physical, chemical and biological properties, studies on the synthesis, characterization, applications and assessments of these materials have promoted the scientific advancement to grow and alter the entire agrifood area [7,8]. Specifically, many reports have focused on the potential applications of nanomaterials as participants to assure food quality, improve packaging and produce food products with altered function and nutrition [1,4,5,9,10].

Packaging is a key component of each stage in the food industry; however, its permeable nature is the major defect in conventional food packaging materials. There are no packaging materials fully resisting water-vapors and atmospheric gases [2]. Moreover, participants along with the food supply chain seek novel, cost-effective, eco-friendly and resourceful food packaging systems to protect and monitor the quality of packaged foods, which is made possible with committed food safety, quality and traceability. As a result, there are several critical factors driving the innovation of food packaging materials to be continuously excavated. On the one hand, food packaging facilitates storage, handling, transport and protection of food from environmental pollution and other influences, and meets the increasing demands of the market, especially related to consumer preference for nutritious and high-quality food products [11]. Some bionanocomposites materials are designed to improve the functional characteristics of general food packaging, such as barrier performance, mechanical strength and thermal stability, and other nanomaterials can incorporate bacteriostatic agents, antioxidants, plant extracts and enzymes to lengthen shelf-life of food products [12]. On the other hand, to date, the majority of materials used in packaging industries are non-biodegradable petroleum-based plastic polymer materials (approximately 8% of the global gas production and fossil feedstock is used to yield synthetic polymers), which in turn, denote a serious problem on the universal environment [13]. The advancement of renewable or green packaging has potentials to reduce the negative environmental impacts caused by the synthetic packaging by using biodegradable or edible materials, plant extracts, and nanocomposite materials [11]. The following two types of materials [14,15,16,17,18,19] are in focus: (1) inorganic and metal nanoparticles (nano-clay, montmorillonite nanoparticles, halloysite nanotubes, AgNPs, ZnO-NPs and CuO-NPs, et al.); (2) plant extracts (milk thistle extract, green tea extract, etc.) mixtures incorporated in biopolymers (chitosan, cellulose, starch, etc.).

Furthermore, the enormous potential of nanotechnology has received attention from researchers in multi-disciplinary areas to develop promising and desirable materials in food packaging systems. On the whole, the applications of nanocomposite materials for food packaging reported in the recent three years are divided into three main functions, i.e., improved, smart and active food packaging [2]. Firstly, improved packaging is that the utilization of nanoparticles in the bionanocomposite materials improves their mechanical and barrier properties, including elasticity, gas barrier characteristics (barrier against oxygen, carbon dioxide, and flavor compounds diffusion) and stability under different temperature and moisture conditions [12]. Secondly, smart (intelligent) packaging performs in terms of information feedback and marketing on real-time quality of packaged food products and also performs as a guard against fraud and fake products and an indicator of the situation of exposure to certain adverse factors such as insufficient temperatures or high oxygen levels [20,21]. Thirdly, active packaging offers protection and preservation grounded on mechanisms activated by inherent and/or acquired factors (antimicrobial activity, biodegradable activity), and achieves the reduction in loss of food products due to extension of their shelf-life [22]. Though there have been considerable studies on novel nanomaterials applications in food packaging reported every day, most materials are still in the stage of feasibility and demonstration studies, and employments in food packaging field are yet to receive approval concerning their safety issues, which could be caused by the migrations of nanomaterials from packaging to food matrix [23]. Moreover, the absorption, distribution, metabolism and excretion as well as toxicological assessment of nanoparticles in food intake of humans are important research focuses [24]. Thus, as can been seen, the use of nanomaterials in the food industry opens up multiple possibilities originating from the inherent features of nano-additives, which are either an improvement of the original polymer properties (e.g., barrier or mechanical properties) or introduction of new functionalities (e.g., active and bioactive packaging or sensing and monitoring). This is an emerging and evolutionary area involving multidisciplinary studies.

This review references more than 170 articles published in the recent three years and summaries the up-to-date developments of nanomaterials applied in the food packaging field, presenting a comprehensive review of various nanostructures and related technologies used to construct functional food packaging systems. The contents of this article mainly concentrate on synthesis methods, physical and chemical properties and biological activity, applications in food systems and safety assessments of different types of nanomaterials. This review also highlights the possible mechanism of some characteristics, such as antimicrobial activity against bacteria and improved reduction and stabilization properties of certain active nanomaterials. In the last part, an outlook of the nanomaterials functionalized in food packaging is included.

## 2. Inorganic and Metal Oxide Nanomaterials Applied in Food Packaging

Generally, nanomaterials applied in food packaging can be classified into two categories: inorganic and organic materials. For the former materials, metals and metal oxides and clay nanoparticles incorporated into bionanocomposite films and nanofibers can be considered [25,26]. Besides common bacteriostatic silver nanoparticles, some of the inorganic agents, like oxidized nanoparticles including CuO, ZnO, TiO_2_, MgO and Fe_3_O_4_, have attracted great interest due to their resistance to the rough processing conditions and enhancement of strong inhibition against foodborne pathogens. As for the other materials like various clays, they could offer resistance to gases and water vapor, and improve the mechanical strength of biopolymers [2,27]. The second group is organic materials including, but not limited to, phenols, halogenated compounds, quaternary ammonium salts, plastic polymers, plus natural polysaccharide or protein materials such as chitosan, chitin, zein and whey protein isolates, which have lately been highly regarded [28,29].

### 2.1. Silver-Based Nanoparticles

So far, in all kinds of nanoparticles developed and characterized, silver-based nanoparticles (NPs) have taken an important place due to their inherent feature of antimicrobial activity even in solid-state samples, and have therefore been used as bacteriostatic agents from ancient times. Silver-salts materials also have an inhibition effect on the growth of diverse pathogens affecting human health, such as those in films, catheters, burns, cuts and wounds to protect them from infection [7]. Silver-based particles in nanoscale include silver nanoparticles (AgNPs), silver nanocluster (AgNC) and silver-based alloy materials [30,31].

#### 2.1.1. Synthesis Methods

AgNPs is one of the most studied and applied antimicrobial agents because of its broad-spectrum antimicrobial activity against microorganisms. The traditional solvothermal synthesis methods of AgNPs-functionalized packaging materials which usually require physical and chemical preparations of synthesizing and immobilizing, however, seem to be very expensive and hazardous and not environmentally friendly. This method has been gradually discarded for the tedious and complicated procedure. Interestingly, AgNPs prepared through biological synthesis exhibit high solubility, yield and stability. Additionally, it is simpler, faster, more environmentally friendly and dependable, and is recognized as a green approach to produce AgNPs with well-defined morphology and size under optimal conditions in favor of application in food packaging [32]. Chu et al. prepared antimicrobial active poly (lactic acid) (PLA)-based films with alloy of AgNPs and zinc oxide nanoparticles (ZnO-NPs) through a solvent volatilizing method [33]. Tao et al. developed a convenient and efficient biosynthesis method to synthesize AgNPs-silk/poly (vinyl alcohol) (PVA) bionanocomposite film by blending AgNPs with PVA [34]. Shao et al. reported a new green chemistry synthetic method of sodium alginate-AgNPs composite by using sodium alginate as a stabilizing agent and ascorbic acid as a reducing agent [35]. Narayanan and Han presented an immobilization method of borate-stabilized AgNPs as nanofillers in dual-crosslinked polymers comprised of PVA and sodium alginate at different ratios [36]. Patra et al. produced a phyto-mediated biosynthesis of AgNPs through utilizing the water extract of watermelon rind under light exposure at room temperature, obtaining prepared AgNPs with an average size of 110 nm and surface plasmon resonance of 425 nm [37]. Azlin-Hasim et al. studied the capacity of a layer-by-layer strategy to prepare low-density polyethylene (LDPE) active films with silver nanoparticles coated for food packaging applications [38]. It is found that the green chemistry synthesis for the silver-based nanoparticles is highly effective and displays high potentials.

#### 2.1.2. Physical, Chemical Properties and Biological Activity

The physical and chemical properties of nanoparticles are important for their action, efficacy, bio-distribution and safety. Accordingly, characterizations of nanomaterial are crucial to evaluate functions of the developed particles [32]. Characterizations are performed using a group of analytical techniques, including transmission electron microscopy (TEM), scanning electron microscopy (SEM), UV-Vis spectroscopy, Fourier transform infrared spectroscopy (FTIR), X-ray diffractometry (XRD), X-ray photoelectron spectroscopy (XPS), dynamic light scattering (DLS), atomic force microscopy (AFM), thermogravimetric analysis (TGA) and differential scanning calorimetric (DSC), to investigate their physical and chemical properties. Those properties include size and size distribution, surface chemistry, particle morphology, coating/capping, particle composition, agglomeration, dissolution rate, thermo-mechanical behavior, rheological property and particle reactivity in solution. It is equally important that the biological activities of nanomaterials are to be examined for ensuring their claimed antimicrobial property and safety concerns. Tao et al. found that PVA film coated by AgNPs-silk showed superior stability, mechanical performance and good antimicrobial activity inhibiting both Gram-positive and Gram-negative bacteria [34]. Arfat et al. developed the bionanocomposite films based on fish skin gelatin and bimetallic Ag-Cu nanoparticles (Ag-Cu NPs). The films were characterized to have improved mechanical property and low transparency, thermal stability, yellowness and high antibacterial activity against both Gram-positive and Gram-negative bacteria [39]. Jafari et al. studied the effect of chitin nanofiber on the morphological and physical properties of chitosan/silver nanoparticle bionanocomposite films, and concluded that AgNPs had dramatically improved the barrier and mechanical properties, but showed a negative impact on color properties [40]. Ramachandraiah et al. demonstrated a higher antioxidant activity of the biosynthesized AgNPs from persimmon byproducts and incorporation in sodium alginate thin films [41].

#### 2.1.3. Applications in Food Systems

Because of the aforementioned unique properties, AgNPs have been widely used in the health care industry, house-hold utensils, food storage, environmental and biomedical applications. Herein, it is interesting to emphasize the applications of AgNPs in food systems, including antibacterial, antifungal, antioxidant, anti-inflammatory, antiviral, anti-angiogenic and anti-cancer. Heli et al. reported that the exposure of corrosive vapor (ammonia) remarkably reduced the population density of AgNPs embedded into bacterial cellulose, causing a large distance between the residual nanoparticles and a decrease in the UV-Vis absorbance related to the plasmonic properties of AgNPs [42]. This material exhibited color changes from amber to light amber upon corrosive vapor exposure, and from amber to a grey or taupe color upon fish or meat spoilage exposure, which opened up an innovative approach and capability in gas sensing to act as a smart packaging for monitoring fish or meat spoilage exposure. Tavakoli et al. investigated the effect of nano-silver packaging in increasing the shelf-life of nuts in an in vitro model, showing an important effect on extending the shelf-life of nuts with the highest shelf-life of hazelnuts, almonds, pistachios and walnuts extended to 18, 19, 20 and 18 months, respectively [43]. Deus et al. evaluated the effect of an edible film coated with nano-silver on the quality of turkey meat during modified atmosphere and vacuum-sealed packaging for 12 days of storage [44]. Ahmed et al. created PLA composite films by loading bimetallic silver-copper nanoparticles and cinnamon essential oil into polymer matrix through compression molding technique, which was utilized in the chicken meat packaging, revealing a new direction of active food packaging to control the pathogenic and spoilage bacteria related to fresh chicken meat [45].

#### 2.1.4. Safety Assessments

On account of gaps in understanding toxicology of nanomaterials, the development of their applications is related to safety concerns. In case of food contact bio-nanocomposite materials, the first steps of consumers’ exposures are the migrations of nanoparticles from packaging to food products. Thus, in order to estimate the risk, we need to know the possibilities of nanoparticles released from food contact materials [46]. Gallocchio et al. evaluated silver migration from a commercially available food packaging containing AgNPs into chicken meatballs under plausible domestic storage conditions, and tested the contribution of this packaging to restrict food spoilage bacteria proliferation [47]. The results showed that the migration was slow and no significant difference in the analyzed bacteria levels between meatballs stored in AgNPs plastic bags and control bags. Tiimob et al. tested the release of eggshell-silver tailored copolyester polymer blend film exposed to water and food samples by atomic absorption spectroscopy (AAS) analysis, showing that AgNPs was not released in chicken breast or distilled water until 168 and 72 h, respectively [48]. Su et al. estimated the effects of organic additives (Irganox 1076, Irgafos 168, Chimassorb 944, Tinuvin 622, UV-531 and UV-P) on the release of silver from nanosilver-polyethylene composite films to an acidic food simulant (3% acetic acid) by detection using inductively coupled plasma mass spectrometry (ICP-MS) and found that additives influenced silver release through two synchronous processes: (1) reactions between silver and organic additives promoted release of silver from the composite film to an acidic food simulant; (2) inhibition or promotion of silver release was influenced by silver oxidation [49]. High humidity and temperature treatment of the prepared films were suggested to markedly enhance silver release by promoting oxidation. Hosseini et al. measured the migration of silver from AgNPs polyethylene packaging based on titanium dioxide (TiO_2_) into Penaeus semisulcatus by a titration comparison within the other migrations, and found that titration had a superior sensitivity compared to the other migration methods in determining the residues of nanoparticles (*p* < 0.05) [50]. Hannon et al. determined the silver release from an experimental AgNPs spray coated on the surface of polyester and LDPE packaging material into milk [51]. The test of coating process suggested the process modification has the potential to reduce migration. Becaro et al. evaluated the genotoxic and cytotoxic effects of AgNPs (size range between 2 and 8 nm) on root meristematic cells of Allium cepa (A. cepa) [52]. The related studies often concentrate on the inhibition of growth of harmful bacteria. Interestingly, Mikiciuk et al. reported that the concentration and type of AgNPs solutions had an important effect on probiotic bacteria [53]. These bacteria were isolated from fermented milk products beneficial for the digestive system, including *Lactobacillus* acidophilus LA-5, *Bifidobacterium* animalis subsp. lactis BB-12 and *Streptococcus* thermophilus ST-Y31, which deserves great public attention.

### 2.2. Zinc Oxide Nanoparticles

Zinc oxide (ZnO) has attracted great interest worldwide because of its excellent properties, particularly resulting from the realization of the development of nanomaterials. Considerable studies of ZnO-NPs have been triggered on the production of nanoparticles using different synthesis methods and on their future applications, attributed to their high luminescent efficiency with a large exciton binding energy (60 meV) and a wide band gap (3.36 eV) [54]. ZnO-NPs usually act as antimicrobial and UV-protective agents used in the food packaging area. The increasing focus on ZnO-NPs drives the innovative development of synthesis methods of nanoparticles and their functions (Table 1).

#### 2.2.1. Synthesis Methods

Synthesis methods of zinc oxide have been developing rapidly. Because the synthesis approach determines the properties of nanomaterial, the selection of synthetic methods is a crucial step in the engineering of ZnO-NPs for a decided utilization. In recent decades, three main approaches have been used for forming ZnO-NPs: physical, chemical and biological methods. Among them, the casting method followed by solvent evaporation is the most common method used in preparation of ZnO nanocomposites with different morphologies. Rokbani et al. reported a synthesis method using a combination of ultrasound stimulations and autoclaving to prepare electrospun nanofibers of mesoporous silica doped with ZnO-NPs [55]. Jafarzadeh et al. used the solvent casting method to prepare nanocomposite films of nano-kaolin and ZnO nanorod (ZnO-nr) complex embedded into semolina film matrices [56]. Youssef et al. prepared a novel bionanocomposites packaging material using carboxymethyl cellulose (CMC), chitosan (CH) and ZnO-NPs by the casting method [18]. Salarbashi et al. developed a soluble soybean polysaccharide (SSPS) nanocomposite incorporating ZnO-NPs using a solvent-casting method [57]. Shahmohammadi and Almasi obtained bacterial cellulose-based monolayers and multilayer films with 5 wt% ZnO-NPs incorporated by using ultrasound irradiation (40 kHz) during ZnO-BC nanocomposites preparation [58]. Akbariazam et al. prepared a novel bionanocomposite of soluble soybean polysaccharide (SSPS) and nanorod-rich ZnO by the casting method [59].

#### 2.2.2. Physical and Chemical Properties and Biological Activity

Compared with traditional antimicrobial agents, metal oxide nanoparticles show higher stability under extreme conditions with antimicrobial activity at low concentrations, and are considered to be non-toxic for humans [54]. Among these metal oxide materials, ZnO-NP is a strong antimicrobial agent [60]. ZnO-NPs exhibited diverse morphologies and showed robust inhibition against growth of broad-spectrum bacterial species. Mizielinska et al. studied the effect of UV on the mechanical properties and the antimicrobial activity against tested microorganisms of PLA/ZnO-NPs films [61]. They found that a decrease in Q-SUN irradiation to the antimicrobial activity of films with ZnO-NPs against *B. cereus*, whereas Q-UV and UV-A irradiation showed no effect on the mechanical properties of developed nanomaterial. Kotharangannagari and Krishnan studied the shape memory properties of novel biodegradable nanocomposites made of starch, polypropylene glycol (PPG), lysine and ZnO-NPs [62]. The results showed shape memory properties in the prepared nanocomposites by treating the sample at 25 °C and then at 55 °C. Furthermore, the mechanical properties showed an increase with increasing of ZnO-NPs content. Babaei-Ghazvini et al. investigated the UV-protective property of the prepared biodegradable nanocomposite films incorporated by starch, kefiran and ZnO-NPs, with a function of ZnO-NPs at different contents (1, 3, and 5 wt%) [63]. The tensile strength and Young’s modulus of the specimens were measured and found that they were increased with Zn content up to 3 wt%, whereas elongation at break of the material was decreased. Besides, it is indicated that an increase of Tm following with Zn content increased thermal properties. Mizielinska et al. reported a test of change in adhesiveness of fish samples stored in fillets in active coating boxes [64]. The result showed a decrease of adhesiveness of the fish sample when stored in an active container. Besides, it was found that packaging materials containing ZnO-NPs were more active against cells of psychotropic and mesophilic bacteria than the coatings with polylysine after 144 h and 72 h of storage. Calderon et al. developed a Zn-ZnO core-shell structure and explored the oxidation capability of carbon supported Zn nanostructures used as oxygen scavenging materials activated by the relative humidity in the environment [65].

#### 2.2.3. Applications in Food Systems

ZnO-NPs are recognized as inexpensive with potential antimicrobial properties. So the applications of ZnO-NPs packaging in food systems concentrate on its antibacterial effect, and they are used to prolong the fresh food products’ shelf-life. Youssef et al. used an innovative carboxymethyl cellulose/chitosan/ZnO bionanocomposite film to enhance the shelf-life of Egyptian soft white cheese [18]. Mizielinska et al. compared the impacts of material containing polylysine or ZnO-NPs on the texture of Cod fillets, and found a lowest water loss when the sample was packed with ZnO-NPs, and an increase in the adhesiveness of the fish samples stored in boxes without active coatings, indicating that ZnO-NPs prevented the adhesiveness of food products [64]. Li et al. estimated the influences of ZnO-NPs incorporation into PLA films on the quality of fresh-cut apples [66]. It was found that the most weight loss was observed in nano-blend packaging films compared to the PLA film at the end of storage; however, packaging nanomaterial provided a better maintenance of firmness, color, sensory quality and total phenolic content. It also exhibited a strong inhibition against the growth of microorganisms. Beak et al. proposed that the synthesized olive flounder bone gelatin/ZnO-NPs film showed antimicrobial activity against *L. monocytogenes* contamination on spinach but with no effect on its quality, mainly including color and vitamin C content [67]. Suo et al. found that ZnO-NPs-coated packaging films increased the occurrence of microorganism injury, which was helpful to control pork meat in cold storage [68]. Al-Shabib et al. prepared *Nigella sativa* seed extract-zinc nanostructures (NS-ZnNPs) material and found that NS-ZnNPs showed inhibition effects on the biofilm formation of four food pathogens including *C. violaceum* 12472, *L. monocytogenes*, *E. coli*, *PAO*1, at their sub-inhibitory concentrations [69].

#### 2.2.4. Safety Assessments

ZnO-NPs are utilized as active materials in food packaging, which might bring a potential risk for consumers contacting with this material. This nanoparticle has been demonstrated in in vivo studies that they can access organs through different pathways such as ingestion, inhalation, and parenteral routes [54]. Ansar et al. suggested that hesperidin augmented antioxidant defense with antiphlogistic reaction against neurotoxicity induced by ZnO-NPs, and the enzyme activity enhanced the antioxidant potential to reduce oxidative stress [70]. Senapati et al. evaluated the immune-toxicity of ZnO-NPs in different ages of BALB/c mice after sub-acute exposure, and found that the aged mice were more susceptible to ZnO-NPs-induced immune-toxicity [71]. Meanwhile, information on the amount of ZnO-NPs contained in food packaging and the impacts of their exposure on intestinal function are still insufficient. Moreno-Olivas et al. found that the amount of zinc present in the food was about 100 times higher than the recommended dietary allowance [72]. The effects of ZnO-NP exposure to the small intestine composed of Caco-2 and HT29-MTX cells was investigated in an in vitro model. It was found that Fe transport and glucose transport following ZnO NPs exposure were 75% decreased and 30% decreased, respectively. Also, the ZnO-NPs affected the microvilli of the intestinal cells. Zhang et al. reported the fate of the packaging material of ZnO-NPs on the coating layer incorporated into PLA-coated paper entering into paper recycling processes [73]. The results of mass balance indicated that 86–91% ZnO-NPs ended up in the material stream, mostly incorporated into the polymer coating; however, 7–16% nanoparticles completed in the desired material stream. Furthermore, the nano-coating showed positive impacts on the quality of recovered fiber. Chia and Leong made a surface modification to decrease the toxicity of ZnO-NPs by silica coating and found a significant decrease on the dissolution of ZnO-NPs [74]. They suggested that the coating offered a possible solution to enhance the biocompatibility of ZnO-NPs, which could broaden the applications such as antibacterial agent in food packaging.

### 2.3. Copper-Based Nanoparticles

Copper-based nanoparticles mainly include copper nanoparticles (CuNPs) and copper oxide nanoparticles (CuO-NPs). Most studies focusing on CuO NPs suggest that this material is one of the most-extensively studied metal oxide nanoparticles. The antimicrobial activity is its important feature, thus this material can be used to reduce the growth of bacteria, viruses and fungi. The nano-sized CuO-NPs were allowed to interact with the cell membrane due to their enormous surface area, and then showed an increased antimicrobial effect [26]. CuO-NPs have been applied intensively in chemical engineering and food and biomedical areas, and used as gas sensors, catalysts, water disinfectants, polymer reinforcing agents, and as a material of food packaging, semiconductors, magnetic storage media, solar cells field, emission devices and so on [75]. Consequently, antibacterial activity of CuO-NPs has been widely utilized in the fields of food packaging materials, polymer nanocomposites and water purification.

#### 2.3.1. Synthesis Methods

CuO-NPs have potentials for forming antimicrobial nanohybrids. Almasi et al. claimed that whether the polymer substrate has already exhibited antimicrobial activity or not, the incorporated CuO-NPs could further increase the activity of the two components contained in the nanocomposite [26]. They have fabricated a novel nanocomposite incorporation into CuO-NPs, bacterial cellulose nanofibers and chitosan nanofibers by a chemical precipitation method. Gu et al. introduced a green, facile and low cost biosynthesis of monoclinic CuO-NPs based on an ultrasound method by using the extracts of *Cystoseira trinodis* as an eco-friendly material [76]. Eivazihollagh et al. reported a facile method to synthesize spherical CuNPs in situ templated by a gelled cellulose II matrix under the alkaline aqueous conditions [77]. No more than 20 min, the nanocomposite material was harvested in a one-pot reaction. Castro Mayorga et al. prepared an active biodegradable nanocomposites of poly(3-hydroxybutyrate-co-3-hydroxyvalerate) (PHBV) melt mixed with CuO-NPs in bilayer structures [78]. This bilayer-structural material was made of an active electrospun fibers mat embedded by PHBV18 (18% valerate) and CuO-NPs, and coating onto a bottom layer of concentration molded PHBV3 (3% mol valerate). Gautam and Mishra [79] synthesized Cu-NPs material compositing edible bilayer pocket prepared by heat and NaBH_4_ treated methods to form a heat-sealable casein protein layer laminated with sodium alginate-pectin.

#### 2.3.2. Physical and Chemical Properties and Biological Activity

The properties of the CuO-NPs depend on the synthesis method and they are very important for their applications in various areas, such as food packaging research, which rely on their biological activity. Although the specific mechanism of the antimicrobial effect of CuO nanoparticles is little known, their antimicrobial actions on bacterial cells have been proposed [75]. Beigmohammadi et al. determined the antimicrobial LDPE packaging films incorporating AgNPs, CuO-NPs and ZnO-NPs in testing of coliform amounts of ultra-filtrated cheese [80]. The results showed the number of surviving coliform bacteria declined to 4.21 log CFU/g after storing for 4 weeks at 4 ± 0.5 °C for all three treatments. Almasi et al. found that the antimicrobial activity of CuO-NPs against both Gram-negative and Gram-positive bacteria was inhibited after attachment to bacterial cellulose nanofibers; however, a synergistic action presented between chitosan nanofibers and CuO-NPs on the antimicrobial activity was reinforced [26]. Shankar et al. evaluated the water vapor permeability, barrier property, UV and thermal stability, and antimicrobial activity of the nanocomposite films [81]. The types of polymers used decided the surface morphology of films. The results showed that the addition of CuO-NPs increased the above-mentioned properties, and the films showed antimicrobial activity against *Listeria monocytogenes* and *E. coli*.

#### 2.3.3. Applications in Food Systems

Nanomaterials with various characteristics generated from many polymers constructing copper-based nanocomposites can be used in a variety of applications. The antimicrobial activity of copper-based nanocomposites reveals applications in engineering food packaging, textile industry, medical devices and water decontamination. Some applications in food packaging over the recent three years are presented in Table 2. However, the actual applications in real food samples were little. Gautam and Mishra synthesized copper-based nanocomposite incorporating a pectin layer to enhance its antimicrobial activities [79]. It was applied in packaging coconut oil and then the oxidative stability of oil was investigated during storage. The results showed that its thermal stability was enhanced due to the nanocomposite addition, and antimicrobial activity of heat-treated nanocomposite film was increased compared to the NaBH_4_-treated NPs film against the growth of *E. coli*. Li et al. prepared chitosan/soy protein isolates nanocomposite film reinforced by Cu nanoclusters, and this material showed the improved elongation at break and tensile strength, and higher water contact angle and degradation temperature and decreased water vapor permeation [82]. Lomate et al. developed an LDPE/Cu nanocomposite film in food packaging to extend the shelf-life of peda, which is an Indian sweet dairy product [83].

#### 2.3.4. Safety Assessments

Although copper-based nanocomposites have been applied for diverse purposes, CuO-NPs and copper ions can be released from the packaging materials into the food systems. Little is known on the toxicity of copper-based nanocomposites and more attentions have been concentrated on CuO NPs [84].

### 2.4. TiO_2_ Nanoparticles

Titanium dioxide nanoparticles (TiO_2_-NPs), as among the most explored materials, are considered as valuable metal oxide nanomaterials with thermostability and inertia. The material also has the ability to modify the properties of biodegradable films. Besides, this material has many advantages such as being cheap, nontoxic, and photo-stable. It has been emerging as a superior photo-catalyst material for energy and environmental fields, such as air and water purification, antimicrobial, self-cleaning surfaces and water splitting [85]. TiO_2_-NPs and their applications in the food packaging area have attracted extensive attentions attributed to their antimicrobial activity.

#### 2.4.1. Synthesis Methods

TiO_2_-NPs tend to aggregate, which would possibly influence the function of film properties. Changing the surface properties of TiO_2_-NPs is an alternative method to solve this problem. In a recent study, several ionic surfactants have been used to non-covalently attach to the surface of TiO_2_-NPs, and more innovational nanocomposites have been synthesized to enhance the TiO_2_-NPs dispersion behavior [86]. He et al. fabricated biodegradable fish skin gelatin-TiO_2_ nanocomposite films by a solvent evaporation method [87]. Li et al. proposed a facile and green method to synthesize super-hydrophobic paper by using a layer-by-layer deposition of TiO_2_-NPs/sodium alginate multilayers onto a paper surface and then with colloidal carnauba wax adsorbed [88]. Lopez de Dicastillo et al. obtained TiO_2_ nanotubes by a deposition process of atomic layer covering the electrospun polyvinyl alcohol (PVA) nanofibers at different temperatures to obtain antibacterial nanostructures with a relative high selective area [89]. Nesic et al. prepared eco-environmental pectin-TiO_2_ nanocomposite aerogels by a sol-gel process and followed by drying under the supercritical conditions [90]. Firstly, pectin was dissolved in water and proper amount of TiO_2_ colloid was added; then crosslinking reaction was induced in the presence of zinc ions and tert-butanol [90]. Finally, the gels were subjected to solvent exchange and supercritical CO_2_ drying.

#### 2.4.2. Physical and Chemical Properties and Biological Activity

Considerable studies indicated that addition of TiO_2_-NPs had promoted the suitability of developed films applied in food packaging. The functional properties of these nanomaterials can be tailored by synthesizing composites that combine the properties of the individual component to achieve synergistic effects. Xing et al. investigated the impact of TiO_2_-NPs on the physical and antimicrobial capabilities of polyethylene (PE)-based films. They found that the antimicrobial activity of the films was attributed to the biocidal action of TiO_2_-NPs against bacteria [91]. Roilo et al. prepared bilayer membranes for food packaging applications by depositing TiO_2_-NPs on PLA substrates, cellulose nanofibers and nanocomposite coatings, and found that the addition of TiO_2_-NPs reduced the penetrant diffusivity but did not affect gas barrier performances, as well as slightly decreased the optical transparency [92]. Oleyaei et al. developed TiO_2_-NPs (0.5, 1 and 2 wt%) incorporated into potato starch films [93]. It was found that TiO_2_-NPs enhanced the optical transparency, and slightly increased the tensile strength and contact angle, and significantly declined the water vapor permeability properties, and decreased the elongation at break of the film. Goudarzi et al. produced the eco-friendly starch and TiO_2_-NPs bio-composites at different TiO_2_-NPs contents (1, 3, and 5 wt%), and investigated the mechanical, physical, water-vapor permeability (WVP), thermal properties and as well as UV transmittance of the synthesized nanomaterial [94]. They found that hydrophobicity increased, and elongation at break and tensile energy to break increased, while tensile strength, WVP and Young’s modulus reduced with increasing TiO_2_ content. Abdel Rehim et al. prepared the photo-catalytic paper sheets by adding different ratios of TiO_2_-NPs/sodium alginate nanocomposite [95]. It was found that biopolymer of sodium alginate reduced the negative effect of the photo-catalyst on paper fibers and increased the adhesion of TiO_2_-NPs to them.

#### 2.4.3. Applications in Food Systems

TiO_2_-NPs are antimicrobial agents. When they are irradiated with UV light, many reactive oxygen species are produced that have the ability to kill microorganisms. Moreover, as nano-additives, TiO_2_ can improve the mechanical properties of polymer nanocomposites [92]. Mihaly-Cozmuta et al. synthesized three active papers based on cellulose, mainly containing TiO_2_, Ag-TiO_2_ and Ag-TiO_2_-zeolite nanocomposites (P-TiO_2_, P-Ag-TiO_2_, P-Ag-TiO_2_-Z), which aimed at being applied in bread packaging [96]. The efficiency in the bread storage was compared in terms of nutritional parameters (proteins, total fat and carbohydrates), acidity, and change of molds and yeasts. Li et al. prepared a novel nano-TiO_2_-LDPE (NT-LDPE) packaging. They investigated the effects of NT-LDPE material packaging on the antioxidant activity and quality of strawberries [97].

#### 2.4.4. Safety Assessments

Nanoparticles exhibited an increased surface-to-mass ratio enhancing the reactivity. Moreover, nanoparticles displayed an increased tendency to penetrate the cell membranes and consequently having the potential to transfer through the biological barriers. So far, the health effects of TiO_2_-NPs have been explored basically on their uptake by inhalation. It was concluded by the International Agency for Research on Cancer (IARC) that decided based epidemiological studies to assess whether TiO_2_ dust causing human cancers was inadequate. Evidence for carcinogenicity in experimental animals was sufficient, which was conducted on account of the induction of respiratory tract tumors in rats after prolonged inhalation. Accordingly, TiO_2_-NPs is classified as a Group 2B carcinogen by the IARC [98]. Based on the extensive food-related uses, an increasing attention has been drawn to the risk assessment of TiO_2_-NPs applied in food packaging. Ozgur et al. evaluated the effect of different amounts of TiO_2_-NPs (0.01, 0.1, 0.5, 1, 10 and 50 mg/L) in vitro at 4 °C for 3 h on sperm cell kinematics with the velocities of Rainbow trout [99]. Additionally, oxidative stress markers (superoxide dismutase (SOD) and total glutathione (TGSH)) of the sperm cells were tested after their exposure to TiO_2_-NPs. The results revealed that a statistical significance (*p* < 0.05) presented in the velocities of sperm cells. When concentration of TiO_2_-NPs reached at 10 mg/L, an increased activity of TGSH and SOD (*p* < 0.05) levels were found. Salarbashi et al. prepared biodegradable SSPS nanocomposites consisting of varying ratios of SSPS and TiO_2_-NPs, and found that TiO_2_-NPs existed in plasma membranes of epithelial cell lines after a 10-day exposure to a number of free nanomaterials [100]. However, anti-cancerous and pro-cancerous activities were not determined because this nanomaterial denoted their neutrality in regards to cancer inhibition or promotion in gastrointestinal tracts. Jo et al. evaluated the interactions between TiO_2_-NPs and biomolecules including albumin and glucose [101]. They investigated that those biomolecules altered the physical and chemical properties as well as the consequence regarding TiO_2_-NPs under physiological conditions. It was found that oral absorption of food grade TiO_2_-NPs was slightly higher compared to general grade TiO_2_-NPs; however, these nanoparticles were excreted through the feces. Besides, the biokinetics of food grade TiO_2_-NPs were extremely relied on their interaction with biomolecules.

### 2.5. Other Metal Oxide and Nonmetal Oxide Nanomaterials

In addition to the metal oxides mentioned above, several other metal oxide and nonmetal oxide nanomaterials also showed an increased potential using as packaging materials, such as MgO-NPs, Fe_3_O_4_-NPs and iron-based nanoparticles, as well as SiO_2_-NPs [102,103,104,105].

MgO naturally exists as a renewable, colorless, crystalline mineral and is economically produced on a large scale. The use of MgO has been recognized as generally safe even in the food applications by the U.S. Food and Drug Administration (FDA). Swaroop and Shukla produced films by incorporating MgO-NPs in PLA polymer through a solvent casting method, and found 2 wt% amount of MgO-NPs in PLA films exhibited the most observed improvement in the oxygen barrier and tensile strength properties, as well as a superior antibacterial efficacy; whereas, nearly a 25% negative effect was found on water vapor barrier properties [102].

Ren et al. synthesized inorganic materials of magnetic ferroferric oxide nano-particles in-situ coating on graphene oxide nanosheets (Fe_3_O_4_@GO) as fillers and then were used to fabricate a PVA nanocomposite film [106]. This material showed a superior barrier capability regarded as a better choice compared to the traditional aluminum films. Shariatinia and Fazli prepared a thickness of 0.13–0.2 mm nanocomposite film made of starch, chitosan, cyclophosphamide, glycerin and Fe_3_O_4_-NPs [103]. Khalaj et al. prepared nanocomposites of the nano-clay containing iron nanoparticles (Fe-NPs)-polypropylene (PP) by a melt interaction. They investigated the morphological, mechanical, gas barrier and thermal properties [107].

The activities of SiO_2_-NPs are related to their average particle size, biocompatibility, high surface area, stability, low toxicity, bad thermal conductivity and supreme insulation [108]. Mallakpour and Nazari developed a facile and fast method to synthesize polymer-based nanocomposite films of PVA and SiO_2_-NPs coating on bovine serum albumin (PVA/SiO_2_@BSA) using a casting method assisted by sonication [108]. Guo et al. investigated the impacts of realistic doses in physiological terms of SiO_2_-NP on gastrointestinal function and health, based on an in vitro model composed of HT29-MTX and Caco-2 co-cultures representing goblet and absorptive cells, respectively [109]. The results showed that the exposure of SiO_2_-NPs was harmful to the brush border membrane and that exposure to the physiologically relevant doses of well-characterized SiO_2_-NP for acute (4 hours) and chronic (5 days) time periods eventually led to adverse effects in cells.

### 2.6. Nano-Clay and Silicate Nanoparticles

At the present time, nano-clay has approximately 70% market value all over the world, meaning that it is the most commercially applied nanomaterial. There are various kinds of clay minerals according to their structures and chemistries as well as sources. Based on the layered structures, these materials are categorized into four major classes, i.e., chlorite, montmorillonite (MMT)/smectite, illite and kaolinite [110]. MMT is recognized as the most commonly used in the preparation of nanocomposites among these clays. The widest acceptability in layered clay is obvious since it has high surface reactivity and surface area [27]. As a result, many studies demonstrated that natural biopolymer-layered silicate nanocomposites significantly improved properties in packaging. In spite of this, there were fewer studies of orgnoclays as nanomaterials in food packaging compared to other nano-encapsulation systems [111].

#### 2.6.1. Synthesis Methods

Halloysite nanotubes (HNTs), as a kind of natural nanomaterials belonging to kaolinite, have a hollow tubular-like structure within the inner and outer diameters of 15 nm and 50 nm, respectively. Due to this tubular shape, HNTs have a capability of being loaded by various materials, which have been developed as functional nanocapsules [112]. Biddeci et al. prepared a functional biopolymer film by filling a pectin matrix modified with HNTs containing peppermint oil, where HNT surfaces were functionalized with cucurbit uril molecules with the aim to enhance the affinity of the nanofiller towards peppermint oil [16]. Pereira et al. prepared lycopene and MMT-NPs in whey protein concentrate films using the casting/evaporation method [113]. Recently, several agricultural processing wastes have been used to synthesize the nanocomposites as raw materials. Orsuwan and Sothornvit developed a biopolymer film incorporated with banana starch nanoparticles (BSNs) and MMT-NPs, where the BSNs was fabricated using miniemulsion cross-linking to make an enhanced agent [114]. Oliveira et al. used the pectin extracted from pomegranate peels to prepare films with the some amounts of MMT-NPs as reinforcement nanomaterial [115]. Zahedi et al. investigated a novel casting method to fabricate a carboxymethyl cellulose (CMC)-based nanocomposite films containing MMT (5 wt%) and ZnO-NPs (1, 2, 3 and 4 wt%) and found addition of ZnO-NPs enhanced the UV-light blocking (from 60% to 99%) of single-layer nano-clay [116].

#### 2.6.2. Physical and Chemical Properties and Biological Activity

Nano-clays, especially MMT, act as crucial fillers in the biodegradation of nanomaterials when incorporated with a polymer, because they are toxin-free, environmentally friendly and safe to be used in food packaging. Besides, their activities to reduce permeability of gases and improve mechanical properties have been confirmed for polymer nanomaterials. Kim et al. investigated a potential application of multilayer packaging films for packing food containing waterborne content, which were prepared by dry laminating commercially available PVA/vermiculite nanocomposites [117]. They found a reversible regression of the barrier properties of oxygen presented in the prepared films. Pereira et al. characterized the structural and mechanical properties of lycopene/MMT/whey protein concentrate films and found that MMT at the amount of 20 g/kg in the polymeric matrix increased both thermal and mechanical properties [113]. Besides the red coloring ability, lycopene showed no effects on detectable interference in the physical or structural properties. Beigzadeh Ghelejlu et al. prepared nano-clay nanocomposite/chitosan active films incorporated with three levels of Silybum marianum L. extract (SME) (0.5% *v/v*, 1% *v/v* and 1.5% *v/v*) and MMT (1, 3 and 5 wt% of chitosan) [19]. The results indicated that the addition of SME and MMT improved the antioxidant properties of the films, but decreased the solubility and WVP and influenced the optical and mechanical properties of films. Notably, plant essential oils have been encapsulated into nano-clay or MMT-NPs to improve the antioxidant and antimicrobial activities of composite materials applied in packaging system, including peppermint, thyme and cinnamon [16,17,118]. Khalaj et al. found that in the prepared nanocomposite of Fe/MMT/PP-NPs, the intercalation and exfoliation of the clay were affected reversely after the addition of PP-NPs to some extent [107]. Furthermore, certain homogeneity of uniform distribution of MMT and PP-NPs was observed through TEM and SEM. The melting temperatures increased with clay concentration; however, crystallization temperature and crystallinity decreased with the clay concentration with NPs compensating the effect of clay. Nano-clays also have prospects in active and intelligent food nano-packaging. Gutiérrez et al. developed a nano-clay of MMT containing blueberry extract [111]. They revealed that according to a shift between flavylium and quinoidal forms of anthocyanins in blueberries, the color was changed following the pH of the system. Thus, addition of blueberry extract could modify the structure of MMT to form novel nano-clays with more active properties.

#### 2.6.3. Applications in Food Systems

Generally, clays are low-cost, naturally occurring and eco-friendly agents and used in various applications. Clay minerals are used in the fields of agriculture, geology, engineering, construction and process industries [27]. Peter et al. investigated the chemical and microbiological characteristics of white bread during the storage in paper packaging modified with Ag/TiO_2_-SiO_2_-NPs [119]. The results showed good water retention and prolonged shelf-life of bread for 2 days compared to the unmodified packaging. Nalcabasmaz et al. developed nanocomposite materials containing 1% nano-clay and 5% poly-beta-pinene (P beta P) [120]. They tested the material for packaging sliced salami. The packaged food sample used nanofilms and multi-layered film under different conditions of vacuum, modified atmosphere packaging of 50% CO_2_ and 50% N_2_ and air, and both stored at 4 °C for 90 days. It was found that the moisture content and hardness showed no significant changes during storage. The sliced salami stored under vacuum and high CO_2_ using the multilayer material displayed the longest storage time of 75 days. Kim et al. developed insect-proof HNTs material, which were applied to a LDPE-based film to control *Plodia interpunctella* (Indian mealmoth) from infesting the food [121]. Peighambardoust et al. prepared LDPE-based Films incorporating with organic clay nanoparticle including cloisite 30B, cloisite 20A and cloisite 15A for packaging to decrease the growth of coliform bacteria in ultra-filtrated cheese [122]. The developed films exhibited a decrease of coliform load to 2.05 log CFU/g at the optimum condition, which was corresponding to Japanese industrial standard (JIS Z 2801:2000). Echeverria et al. evaluated the future application of active nanocomposite films based on soy protein isolate-MMT loaded with clove essential oil to preserve the muscle fillets of Bluefin tuna stored in refrigerator [123]. They further analyzed the possibility of clay in packaging diffusing to the food system. Clay inclined to release the clove oil by extending its antimicrobial activity (especially against *Pseudomonas* spp.) and enhancing antioxidant activity. There were no observed metals (Si and Al) of clay diffused to the muscle of fish. Guimaraes et al. evaluated fresh-cut carrots (FCC) coated by MMT-NPs subjected to packaging of passive modified atmosphere [124]. The use of starch nanoparticles incorporated into coating film together with a modified atmosphere led to the enhanced total antioxidant activity, volatiles, and organic acids maintaining of FCC. Junqueira-Goncalves et al. evaluated the effect of addition of MMT-NPs to a lacto-biopolymer coating [125]. They found that the material could improve its water vapor barrier and reduce weight loss, as well as oxygen uptake and the release of carbon dioxide, and improve fruit firmness and reduce mold and yeast load, at last prolong the shelf-life of coated strawberries.

#### 2.6.4. Safety Assessments

With nano-clay or MMT-NPs materials attracting more and more attentions worldwide, analyses of risks of these nanomaterials to the lung health of exposed workers have been emerging. Besides, present studies aiming to demonstrate the toxicological actions of nano-clay showed that the structure had resulted in the promotion of cellular uptake and interactions [126]. Han et al. presented a study on the degradation and release of nano-clay-loaded LDPE composite for food packaging [127]. It was found that the toxicity of released nano-clay particles from nano-clay particle-embedded LDPE composites to A594 adenocarcinomic human alveolar basal epithelial cells was degraded. Wagner et al. investigated the potential of Cloisite 30B and Cloisite Na^+^ and their thermally degraded byproducts and then induced toxicity in the model of lung epithelial cells of human [126]. Analysis of byproduct physical and chemical properties suggested changes happened in structures and functions. Echegoyen et al. investigated the migration of nano-clay from food packaging materials to food samples [128]. The results showed that Al-NPs of different sizes and morphologies could migrate into food stimulants with different food stimulants (acetic acid 3% and ethanol 10%), temperatures and times (70 °C for 2 h and 40 °C for 10 days) from two commercialized LDPE-based nanocomposite bags analyzed by ICP-MS.

## 3. Organic Biopolymer-Based Nanomaterials Applied in Food Packaging

The concept of a bio-based economy is gradually receiving attentions from scientific, societal, and economic aspects, and there is a great deal of driving force to develop strategies for this purpose [129]. The inspiration of producing biopolymer-based materials is to utilize renewable organic sources, including polysaccharides and proteins, aiming at replacing non-renewable fossil sources. There are various organic nanomaterials applied in food packaging, mainly divided into three categories: polymer-based plastics, polysaccharide-based and protein-based nanomaterials, which provide biopolymer matrix for nanocomposite materials.

### 3.1. Polymer-Based Nanomaterials

Traditionally, most plastic packaging materials are made from petroleum-based polymers, mainly containing the commodity polystyrene (PS) and polyethylene (PE), which are recognized as not being environmentally friendly. With the development of new polymer materials, the biodegradable polymer-based plastics can provide a viable alternative, such as PVA [130,131,132,133,134], polylactic acid (PLA) [135,136,137,138,139], poly(3-hydroxybutyrate) (PHB) [140,141] and poly(3-hydroxybutyrate-co-3-hydroxyvalerate) (PHBV) [78,142] and their biopolymer blends.

#### 3.1.1. PVA

PVA, basically made from polyvinyl acetate through hydrolysis, is easily degraded by biological organisms in water. It has been extensively incorporated into other polymer-based compounds to increase the mechanical properties attributed to its hydrophilic properties and compatible structure, including mechanical performance, solvent resistance, biocompatibility and high hydrophilicity [143]. Yang et al. prepared chitosan/PVA hydrogels containing lignin nanoparticles (LNPs) (1 wt% and 3 wt%) by a freezing–thaw procedure [130]. The study of mechanical, microstructural and thermal characterizations of the nanomaterial showed that the optimal amount of LNPs was at 1 wt%, whereas the agglomerates at higher LNP content were formed and affected the properties [144]. Sarwar et al. investigated the impact of Ag-NPs embedded into nanocellulose on the mechanical, physical and thermal properties of PVA-based nanocomposite films [131]. They found that these films had a superior antimicrobial activity against *E. coli* (DH5-alpha) and *S. aureus* (MRSA). Furthermore, the films showed no cytotoxicity effect on HepG2 and the cell viability was above 90%.

#### 3.1.2. PLA

PLA has drawn more attention resulting from the good biodegradability and being a candidate of substitution for traditional polymers. PLA is mainly produced by condensation polymerization from lactic acid, derived from fermentation of corn, sugars, tapioca or sugarcane. Among the various biopolymers investigated, PLA exhibits key properties, including biodegradability, renewability and superior mechanical properties, crystallinity and process ability [102,145]. Aframehr et al. investigated the impact of calcium carbonate (CaCO_3_) nanoparticles on the biodegradability and barrier properties of PLA [137]. The results showed that the barrier properties were increased by loading CaCO_3_-NPs increasing to 5 wt%. It was also found that the gas permeability of CO_2_, O_2_ and N_2_ were enhanced by increasing temperature but decreased by increasing feeding pressure. Vasile et al. prepared the Cu-doped ZnO powder embedded into PLA samples functionalized with Ag-NPs composites by a melt blending process [136]. The results showed an increase of the crystalline degree of PLA when the content of nanoparticle was increased from 0.5 wt% to 1.5 wt%.

#### 3.1.3. PHBV

Polyhydroxyalkanoates (PHAs) have been gradually paid attention recently as biodegradable and biocompatible thermoplastics in packaging applications. The most extensively studied polymer from the PHAs is the poly(3-hydroxybutyrate), PHB, which is partially crystalline with a high rigidity and melting temperature. To decrease the crystallinity, the copolymer obtained with the insertion of 3-hydroxyvalerate (HV) units, named as PHBV, is usually employed with improved handling properties of PHB films [78]. Zembouai et al. prepared blends of PLA and PHBV at different PLA/PHBV weight ratios (0/100, 25/75, 50/50, 75/25, 100/0) through a melt compounding process [146]. The formed blends were investigated on the mutual contributions to flammability resistance, thermal stability, rheological behavior and mechanical properties. The results revealed that increasing PLA content in PLA/PHBV blends led to improved properties, such as flammability resistance and thermal stability. Shakil et al. developed the sepiolite/PHBV nanocomposite films by using the APTES grafted sepiolite through the solution-casting method [142]. The results provided evidence that the application of biodegradable nanocomposite films would lead to a more efficient water barrier and thermal properties.

### 3.2. Polysaccharide-Based Nanomaterials

#### 3.2.1. Starch-Based Nanomaterials

Aqlil et al. investigated a graphene oxide (GO)-filled starch/lignin polymer bionanocomposite [147]. They found that the amount of GO had a strong influence on the mechanical properties and could reduce water vapor permeability and moisture uptake of the prepared film. Shahbazi et al. developed starch film incorporated with multi-walled carbon nanotubes with or without hydroxylation and found that the hydrophobic character of the film was greatly improved with incorporation of a nanotube [148]. Oleyaei et al. estimated the thermal, mechanical and barrier properties of TiO_2_ and montmorillonite on potato starch nanocomposite films [93]. The results showed elongation at break, tensile strength, melting point and glass transition temperature of the films were improved followed the addition of MMT and TiO_2_. The visible, UVA, UVB and UVC lights transmittance and water vapor permeability decreased with the increasing amounts of TiO_2_ and MMT.

#### 3.2.2. Cellulose-Based Nanomaterials

Shankar and Rhim prepared nanocellulose material and tested the effects on the properties of agar-based composite films [149]. The crystallinity index of nanocellulose (NC, 0.71) was decreased compared to the micro-crystalline cellulose (MCC, 0.81). The results demonstrated that NC could be used as an enhanced agent for the preparation of biodegradable composites films. Pal et al. synthesized reduced graphene oxide and cellulose nanocrystal incorporated into PLA matrix through a modified Hummer’s method and an acid hydrolysis [150]. They found that the mechanical property of scaffold was significantly improved. Both tensile strength (23% increase) and elongation at break were increased, which indicated the nanocomposite was ductile compared to unmodified PLA. The distinct anti-bacterial efficacy was observed to inhibit against both Gram-negative *E. coli* and Gram-positive *S. aureus* bacterial strains. Liu et al. prepared starch-based nanocomposite films improved by cellulose nanocrystals to control d-limonene permeability [151]. They found that cellulose nanocrystals amount and aspect ratio were independently controlling d-limonene permeability through film-structure regulation. Lavoine et al. simulated release and diffusion of active substances made of cellulose nanofiber coating to food packaging material through calculating in a mathematical model derived from Fickian diffusion. They found the model was validated for caffeine only [152].

#### 3.2.3. Chitosan-Based Nanomaterials

Postnova et al. studied approaches in which monolithic hydrogels were prepared through mineralization of polysaccharide by a method of green sol-gel chemistry, compared with a method through the formation of polyelectrolyte complex [153]. It was found that both approaches were available for the preparation of films with nanoparticles and chitosan bionanocomposites. Liang et al. prepared edible chitosan films incorporated with epigallocatechin gallate nanocapsules and characterized their antioxidant properties [154]. It was found that the addition of nanocapsules to chitosan hydrochloride films improved their tensile strength, whereas the percent of elongation at break and lightness was significantly decreased. Buslovich et al. developed in situ chitosan and vanillin incorporated on packaging films, containing an aqueous/ethanol solution onto a PE surface by an ultrasonic method [155]. The results showed that increased contact surface strongly inhibited the fruit microbial spoilage.

### 3.3. Protein-Based Nanomaterials

#### 3.3.1. Zein-Based Nanomaterials

Zein, a group of prolamins from corn, is a Generally Recognized as Safe (GRAS) food-grade ingredient. With the hydrophobicity of three quarters of the amino acid residues in zein, zein-based nanomaterials have low WVP compared to many other bio-based films. Moreover, zein-based nanomaterials embedded with inorganic AgNC may have advantages such as low toxicity [30]. Aytac et al. synthesized thymol (THY)/gamma-Cyclodextrin(gamma-CD) inclusion complex (IC) encapsulated electro-spun zein nano-fibrous webs (zein-THY/gamma-CD-IC-NF) as a food packaging nanomaterial and found that zein-THY/gamma-CD-IC-NF (2:1) significantly inhibited the growth of bacteria in meat samples [156]. Rouf et al. prepared nanocomposite with the addition of silicate NPs (Laponite) to zein films casting from 70% ethanol solutions [157]. The changes in the surface energy of the films were evaluated using contact angle measurements and showed an increase in surface hydrophobicity. The Young’s modulus and tensile strength were increased with increasing nanoparticle concentration. The glass transition temperature was increased and WVP was decreased with only a small amount of Laponite. Oymaci and Altinkaya prepared whey protein isolate (WPI)-based films embedded into zein nanoparticles (ZNPs) coated with sodium caseinate by a casting method [158]. They found that the addition of zein NPs dramatically improved the mechanical and water vapor barrier properties of the WPI with no effect on the elongation of the films. It was also found that both the fractional free volume and hydrophilicity of the WPI films decreased. Gilbert et al. prepared a biopolymer-based composite film of hydroxypropyl methylcellulose and ZNPs [159]. The results exhibited an increase in tensile strength, a decrease in elongation, and an initial increase followed by gradual decrease in Young’s moduli with increasing ZNPs.

#### 3.3.2. Whey Protein Isolate-Based Nanomaterials

Qazanfarzadeh and Kadivar prepared WPI-based composite films with different proportions of oat husk nanocellulose (ONC) obtained from acid sulfuric hydrolysis by a solution casting method [160]. They found that the crystallinity increased after acid hydrolysis. The films prepared with 5 wt% ONC showed the highest tensile strength, Young’s modulus, solubility and the lowest elongation at break and moisture content. However, WVP and film transparency were decreased with the addition of ONC. Hassannia-Kolaee et al. prepared whey protein isolate/pullulan (WPI/PUL) films having different contents of nano-SiO_2_ (NS) using a casting method [161]. The results revealed tensile strength of nanocomposite films was enhanced but elongation at break was declined after increasing NS content. Moisture content, water absorption and solubility in water were improved followed as increasing content of NS and the water resistance and barrier properties of the films were also improved. Water vapor permeability of films was decreased with the increasing NS content. Zhang et al. developed a chitosan/WPI film incorporated with TiO_2_-NPs and found that the nanoparticles improved the compatibility of WPI and chitosan [86]. Nanoparticle incorporation increased the whiteness of chitosan/WPI film, but decreased the transparency. The elongation at break and tensile strength of nanocomposite film were increased by 12.01% and 11.51%, respectively, whereas WVP was decreased by 7.60%.

## 4. Mechanistic Studies of Nanomaterials in Food Packaging

Preventing microbial growth in foods is known as a critical function of packaging to meet the challenge of preserving the quality of food products. Accordingly, antimicrobial materials in food packaging are emerging as a promising technology to fulfill the demands. With the applications of antimicrobial agents in food packaging materials, the growth of bacteria is inhibited and thus the shelf-life of food products is prolonged considerably. Antimicrobial materials are grouped into two classes: organic and inorganic materials. Chitosan-based nanoparticles and chitin-based nanoparticles are typical examples of organic materials, which have lately been widely studied. In respect to the organic antimicrobial materials, some noble metals such as Ag-NPs, Cu-NPs and Au-NPs, as well as the oxidized nanomaterials including ZnO, TiO_2_ and MgO have attracted much interest because of their resistance to the rough processing conditions and enhancement of strong biocidal impacts against foodborne pathogens [25].

**Figure 1:**
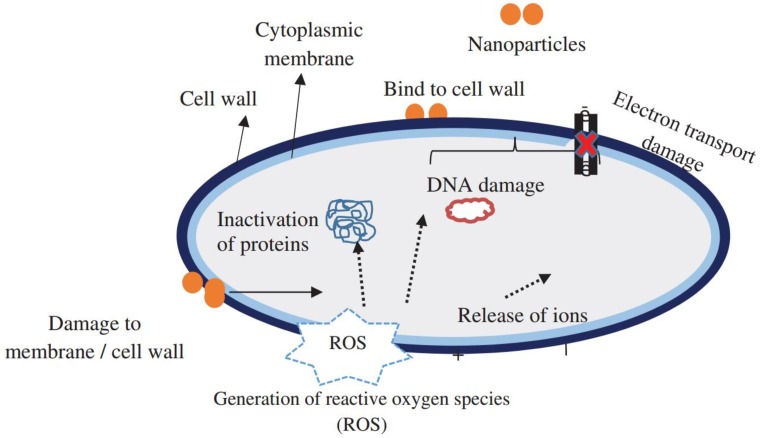
Schematic representation of antimicrobial mechanisms of inorganic nanoparticles. Reproduced with permission from [25]. Copyright Taylor & Francis Online, 2018.

An illustration of reported biocidal mechanisms induced by these nanoparticles is shown in Figure 1 [25]. There are several hypotheses with respect to antimicrobial mechanistic actions of nanomaterials. There was a general consensus that nanomaterials are proved to be an ideal alternative to traditional plastics and they have also served as a potential packaging material to prolong the shelf-life of food products. Because the large surface-to-volume ratio provides more direct interaction to bacterial surfaces, these nanomaterials showed excellent antibacterial properties. Particularly, cationic nanoparticles were firmly attached to the membrane of bacteria with negatively charged outer layers by electrostatic interactions. Disruption of the cell integrity resulted in the leakage of cell contents. Nanoparticles had intrinsic antibacterial activities to refuse the microbes by mimicking natural course of killing by phagocytic cells, i.e., by producing large quantity of reactive nitrogen species (RNS) and reactive oxygen species (ROS). Besides, nanomaterials could also prevent or overcome biofilm formation. Nanoparticles especially metallic nanoparticles exerted toxic effects by enhancing the natural immunity or mimicking natural immune responses by generating a large quantity of RNS or ROS. Others possibly exerted direct killing effects maybe by directly targeting cellular proteins, DNA or lipids [162].

Fardioui et al. reviewed the antimicrobial mechanisms of ZnO-NPs [54]. They found that the explicit mechanisms were still under debate, but several models were suggested as follows: (1) electrostatic interactions between cell walls and ZnO-NPs to destroy bacterial cell integrity; (2) liberation of antimicrobial Zn^2+^ ions regarding accumulation of ZnO-NPs into bacteria cells; (3) ROS formation. Shao et al. tested the electronegativity on *S. aureus* and *E. coli* surfaces after AgNPs treatment, and found a possible change in bacterial surface properties [35]. Furthermore, they observed that cell surfaces were strongly distorted after AgNPs treatment. Meanwhile, some nanoparticles were distributed in the bacterial cell surface, which indicated their direct interaction with bacteria and generation of electronic effects and enhancement of reactivity of AgNPs. El Zowalaty et al. suggested that the chitosan reacted with proteins on microbial surfaces and caused the leakage of intracellular contents [163]. It also chelated trace metal ions and disrupted the electron transport chain. Furthermore, it interfered with the formation of mRNA and proteins once inside the cell [162].

## 5. Certain Aspects of Concerns

In the past decades, nanomaterials in food packaging applications have been developed to enhance the barrier and mechanical properties of traditional and bio-based packaging materials, and/or to provide novel active and smart functionalities. Active and smart packaging materials deliberately incorporate active or smart components, which are intended to release or absorb substances into, onto, or from the packaged food or the surrounding environment, or to provide the intended information of their use conditions [164]. Developments of nanotechnologies are going to increasingly find utilizations in the food packaging area. However, there are gaps in our knowledge on them that put up questions to the scientific community, especially related to toxicity and ecotoxicity. Theoretically, nanoparticles have the potential to migrate to the packed food, but migration assays and risk assessment are still not conclusive [165]. Migrations into food could be considered as the process of mass transfer, in which the low-molecular mass constituents initially existed in the packaging and then released to the packed matrix. Therefore, it was considered as a diffusion process which could be described by Fick’s second law. Thus, one of the most important steps during the development of novel food packaging materials is the research of the migration to investigate the probabilities of any undesirable or harmful ingredients migrating to the food products in overall and specific terms [165].

**Figure 2:**
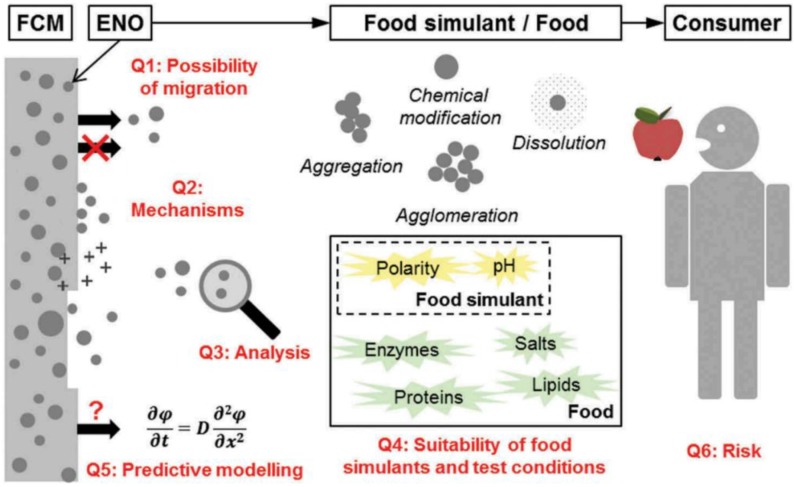
Overview of the six open questions about the migration of NPs from polymer-based food-contact materials identified in Jokar et al.’s review (FCM = food-contact materials, ENO = engineered nano-objects) [166].

Jokar et al. summarized six main questions during the process of migration of NPs from polymer-based food contact materials to consumers in Figure 2 [166].

Briefly, through the investigation, Jokar et al. found that many experimental studies had not given a conclusive answer on the possibility of migration of NPs from food packaging materials to the food products [166]. They assumed this could be partially attributed to the lack of suitable analytical methods for the detection of low quantities and small sizes of NPs. They strongly suggested that studies which concluded that no migration occurred add information about the detection limit of the measurements, including both particle mass or number concentration and particle size. Analytical techniques such as single-particle inductively coupled plasma mass spectrometry (SP-ICP-MS) have been gradually playing a role in characterizing and quantifying NPs in the food simulants extracts [167,168]. Besides, it was difficult to conclude the migrations of NPs through predictive models only considering migrations based on diffusion. Generally, three sub-processes in NP migration could be distinguished: (1) diffusion of the molecule into the polymer to food products because of a concentration gradient; (2) desorption of the molecule from the polymer and subsequent adsorption by the food at the food-packaging interface; (3) diffusion of the molecule in the food due to a concentration gradient [166]. The food stimulants and test conditions would affect the migration and ingestion actions by electrostatic interactions and chemical or mechanical decomposition and organic additives and treatment of food samples [49,169,170]. However, there was a clear lack of data on potential release mechanisms of identified NPs. The question of risk for the consumer associated with migrating NPs from food packaging probably was more complicated than other questions, because there were many influences of physio-chemical characteristics of NPs on gastrointestinal absorption, such as composition, morphology, charge, surface properties and aggregation state and food components [169]. These clearly require more exploration in the future.

In addition to the technical aspects, no regulations on nanotechnology applications have been yet established in a global context mainly because of lack of sufficient and reliable fundamental researches in regard to the safety assessment and migration characters of nanomaterials from packaging to the food system. The FDA considered that evaluations of safety, effectiveness, public health impact, or regulatory status of nanotechnology products should consider any unique properties and behaviors imparted by the application of nanotechnology [171]. The European Commission already edited statutory contents in this direction with technical guidance mentioning nanomaterials, and also recognized active and smart materials and papers to state that new technologies, which are based on engineered materials in nanoparticle sizes that exhibited physical and chemical properties are significantly different from those at a much larger scale. A risk assessment on a case-by-case basis until more is known about the novel technology is needed [172]. On this basis, and taking into account the lack of knowledge about their potential toxicity (oral exposure to nanomaterials had received less attention than the dermal or inhalation pathways [101]), the concept of functional barriers used to prevent migration of contaminants, which were not evaluated by health authorities, could not be applied in the specific case of biopolymer nanocomposites packaging. These statements notably differentiated nano-structure substances from non-nano-structure substances which were authorized for use as a functional barrier, providing that they fulfilled certain standards and the migrations remained below a given limit of detection.

## 6. Conclusions

The innovation of nanomaterials in the food packaging science has brought many changes in food preservation, storage, distribution and consumption. Thanks to preventing microbial growth in foods by antimicrobial activity of nanomaterials, these changes have extended the shelf-life of foods to certain degrees with better management of spoilage in food products. Furthermore, the nanotechnology provides numerous choices for cost-effective, eco-friendly, degradable and renewable packaging materials, which have been gaining more attention and acceptance to solve the ecological environment pollutions and food shortage crises by ensuring food reaches the masses. It is pertinent to note that there are some fundamental studies on toxicity and ecotoxicity, migration assays and risk assessment of nanocomposite materials still needed. In this way, it could allow nanomaterials to work better in the food packaging field.

## Figures and Tables

**Table 1:** Application of Zn/ZnO nanoparticles [54].

**Field of application**	**Example**
Biology and medicine	Bio-imaging
	Drug and gene delivery
	Antitumor and antimicrobial activity
Cosmetic industry	UV filters in sunscreens
	Mineral cosmetics
Manufacturing and materials	Antimicrobial food packaging
	Protection from exposure to UV rays
	Antimicrobial textiles
Energy and electronics	Chemical sensors based on zinc oxide
	Low cost solar cells
	Nano-generator power sensors based on ZnO nanowires

**Table 2:** Main applications of copper-polymer nanocomposites [84].

**Polymer matrix**	**Microorganism**	**Food packaging application**
Cellulose	*S. cerevisiae*	Fruit juices
Hydroxypropyl methylcellulose	*S. epidermis*, *Streptococcus A.*, *E.faecalis*, *B.cereus*, *P. aeruginosa*, *Salmonella*, *Staphylococcusaureus*	Meat
Polylactic acid	*Pseudomonas* spp.	*Not mentioned*
Agar	*L. monocytogenes*, *E. coli*	*Not mentioned*
High density polyethylene	*E. coli DHSα*	*Not mentioned*
